# A Novel Isolation Approach for GaN-Based Power Integrated Devices

**DOI:** 10.3390/mi15101223

**Published:** 2024-09-30

**Authors:** Zahraa Zaidan, Nedal Al Taradeh, Mohammed Benjelloun, Christophe Rodriguez, Ali Soltani, Josiane Tasselli, Karine Isoird, Luong Viet Phung, Camille Sonneville, Dominique Planson, Yvon Cordier, Frédéric Morancho, Hassan Maher

**Affiliations:** 1 Laboratoire Nanotechnologies Nanosystèmes, Institut Interdisciplinaire D’innovation Technologique, Université de Sherbrooke, 3000 Boulevard de l’Université, Sherbrooke, QC J1K 2R1, Canada; zahraa.zaidan@usherbrooke.ca (Z.Z.); nedal.al.taradeh@usherbrooke.ca (N.A.T.); mohammed.benjelloun@usherbrooke.ca (M.B.); christophe.rodriguez@usherbrooke.ca (C.R.); ali.soltani@usherbrooke.ca (A.S.); 2 Laboratoire de Recherche Spécialisé dans L’analyse et L’architecture des Systèmes, The National Centre for Scientific Research, Université Toulouse III Paul Sabatier, 31062 Toulouse, France; josiane.tasselli@laas.fr (J.T.); kisoird@laas.fr (K.I.); 3 Institut National des Sciences Appliquées de Lyon, Ecole Centrale Lyon, The National Centre for Scientific Research, University Claude Bernard Lyon 1, CNRS, Ampère, 69621 Villeurbanne, France; luong-viet.phung@insa-lyon.fr (L.V.P.); camille.sonneville@insa-lyon.fr (C.S.); dominique.planson@insa-lyon.fr (D.P.); 4 Univ. Côte d’Azur, CNRS, CRHEA, rue Bernard Grégory, 06560 Valbonne, France; yvon.cordier@crhea.cnrs.fr

**Keywords:** vertical GaN FinFET, GaN HEMT (high electron mobility transistor), TCAD-sentaurus, isolation, highly doped GaN, integrated circuits

## Abstract

This paper introduces a novel technology for the monolithic integration of GaN-based vertical and lateral devices. This approach is groundbreaking as it facilitates the drive of high-power GaN vertical switching devices through lateral GaN HEMTs with minimal losses and enhanced stability. A significant challenge in this technology is ensuring electrical isolation between the two types of devices. We propose a new isolation method designed to prevent any degradation of the lateral transistor’s performance. Specifically, high voltage applied to the drain of the vertical GaN power FinFET can adversely affect the lateral GaN HEMT’s performance, leading to a shift in the threshold voltage and potentially compromising device stability and driver performance. To address this issue, we introduce a highly doped n^+^ GaN layer positioned between the epitaxial layers of the two devices. This approach is validated using the TCAD-Sentaurus simulator, demonstrating that the n^+^ GaN layer effectively blocks the vertical electric field and prevents any depletion or enhancement of the 2D electron gas (2DEG) in the lateral GaN HEMT. To our knowledge, this represents the first publication of such an innovative isolation strategy between vertical and lateral GaN devices.

## 1. Introduction

Gallium Nitride (GaN) has recently emerged as a transformative material, poised to replace silicon (Si) in a wide range of applications due to its superior physical properties [1]. With its exceptional breakdown field, high saturation velocity, and wide bandgap, GaN-based devices exhibit outstanding performance in high-voltage and high-switching-frequency applications [2,3,4,5]. This has spurred the development of advanced GaN-based components, including GaN High Electron Mobility Transistors (HEMTs) [6,7,8,9] and vertical GaN devices [10,11].

As GaN-based power converters advance, they are projected to dominate the semiconductor market over the next decade, delivering significant economic advantages, particularly in the Electric Vehicle (EV) sector with Power Electronic Conversion Systems (PECSs) [12,13].

In this study, we introduce a pioneering technology that integrates GaN vertical and lateral devices on a single wafer. This novel integration enables the realization of a high-power switch device that is seamlessly combined with a HEMT-based gate driver, thus enhancing the performance and efficiency of power converters.

A critical challenge in this integration is achieving effective electrical isolation between the GaN devices. The vertical GaN power FinFET is constructed on an n-doped GaN substrate, which is also utilized by the HEMT. This configuration exposes the HEMT’s backside to high voltages, potentially compromising its performance [14]. Notably, this high backside voltage can induce significant drift in the threshold voltage (Vth) of the GaN HEMT, leading to instability that adversely affects the gate driver output signal. This instability introduces unpredictable timing delays, undermining the switching performance of GaN power devices and diminishing overall power conversion efficiency, which could even result in device failure.

The impact of the substrate voltage on the threshold voltage of the HEMT device is presented in the literature as a major problem that leads to an instability of the device’s operation mode [15,16]. In this paper, a new approach is proposed to make the device unresponsive to the voltage applied to the back side of the substrate and improve the stability and robustness of the device.

**Figure 1:**
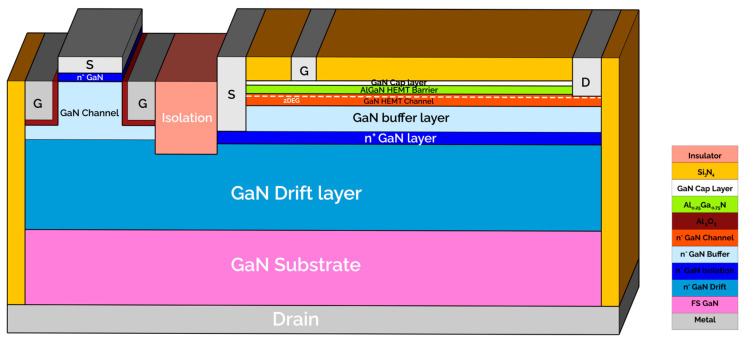
Detailed schematic, cross-sectional illustration depicting the advanced monolithic integration of vertical and lateral GaN-based devices on a unified GaN substrate.

To address this challenge, we utilize TCAD-Sentaurus simulations to investigate a novel isolation technique that separates vertical from lateral GaN transistors on the same wafer (refer to Figure 1). This approach aims to advance the integration capabilities of GaN technology and improve device performance in high-power applications.

## 2. Structure Description

**Figure 2:**
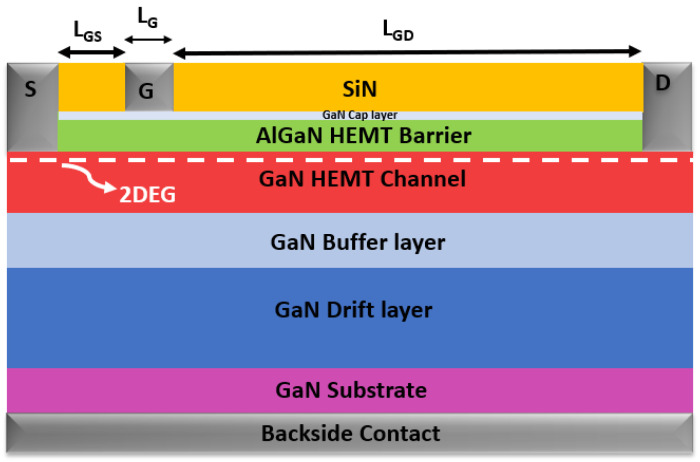
Advanced schematic cross-sectional representation of the AlGaN/GaN HEMT structure, incorporating a strategic backside contact, as implemented in TCAD-Sentaurus simulations.

To elucidate the influence of bias conditions on GaN-HEMT devices integrated alongside high-power vertical devices on the same wafer, an additional ohmic contact has been strategically incorporated into the backside of the GaN-HEMT structure, as illustrated in Figure 2. This fourth contact facilitates the application of varying voltages, thereby replicating the operational scenarios encountered by the vertical device. Under these tailored conditions, the electrical behavior of the GaN-HEMTs was rigorously examined.

The realization of this complex device architecture on actual wafers is achieved through a precise process. Initially, the GaN vertical device’s epi-layer structure is grown using an epitaxy reactor. Subsequently, the designated HEMT fabrication area undergoes etching down to the drift region. Following this, the HEMT epi-structure is selectively grown within the etched region, employing masking techniques to shield the corresponding GaN vertical device epi-layers.

The comprehensive schematic cross-sectional depiction of the AlGaN/GaN HEMT structure utilized in the simulation is provided in Figure 2. The epitaxial structure, which is grown on a n^+^-doped GaN substrate, commences with a 300 nm thick drift layer. This is followed by the active HEMT region, which is composed of a 500 nm thick buffer layer, a 50 nm GaN channel layer, a 20 nm AlGaN layer, and a final 2 nm thick GaN cap layer.

The GaN HEMT model was developed within Sentaurus TCAD through an extensive calibration procedure. This process relied on empirical data obtained from a HEMT device fabricated by Prof. Hassan Maher’s team at Université de Sherbrooke (UdS-LN2). The simulation’s physical parameters for the epitaxial layers were carefully adjusted to closely match the characteristics observed in the actual fabricated devices. For instance, electron mobility was calibrated to 1810 cm^2^/V·s, and the density of deep, single-level trap states was set to 7.8 × 10^12^ cm^−2^. While specific citations are not available in this context, these parameters are in alignment with those documented in the scholarly literature concerning high-performance GaN HEMTs. As an example, Altuntas et al. reported high-quality AlGaN/GaN heterostructures exhibiting impressive electrical properties with a sheet carrier density of 1.28 × 10^13^ cm^−2^ and mobility of 1930 cm^2^/V·s [17], which are comparable to the values used in this study.

The epitaxial layer thicknesses and doping concentrations are detailed in Table 1. The simulation was carried out using the TCAD-Sentaurus Device Simulator by Synopsys Corporation [18]. The calibration of the simulator was based on experimentally measured I–V characteristics from a previously fabricated HEMT device. In this simulation, a reduced thickness of the drift region was used in the vertical device to enhance the effect of the backside electrode voltage on the GaN HEMT device’s performance.

The source-to-gate (L_GS_) and drain-to-gate (L_GD_) extensions used are 0.5 µm and 1.5 µm, respectively, with a gate length (L_G_) of 0.4 µm.

## 3. Results and Discussion

### 3.1. Influence of the High Voltage Applied on the Backside Contact

**Figure 3:**
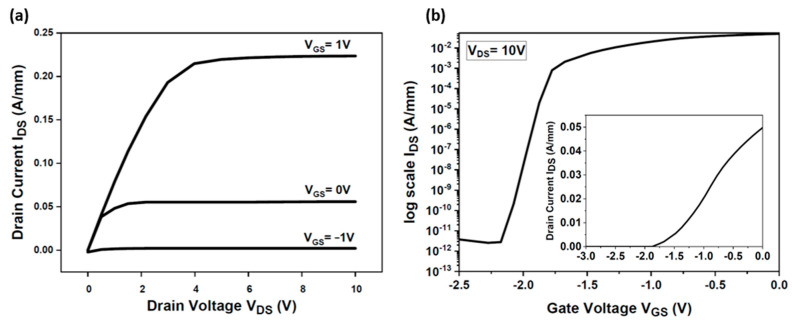
(**a**) I_DS_ (V_DS_) output and (**b**) I_DS_ (V_GS_) transfer characteristics of the GaN HEMT structure simulated by Sentaurus TCAD.

After the calibration phase, the GaN epitaxial structure of the HEMT device was modified by adjusting doping concentrations and layer thicknesses to be compatible with the design for the vertical device structures intended for monolithic integration, as depicted in Figure 1. The outcomes illustrated in Figure 3 correspond to this modified HEMT structure.

The output characteristics depicted in Figure 3 demonstrate the device’s performance across a range of operating conditions, exhibiting good functionality.

**Figure 4:**
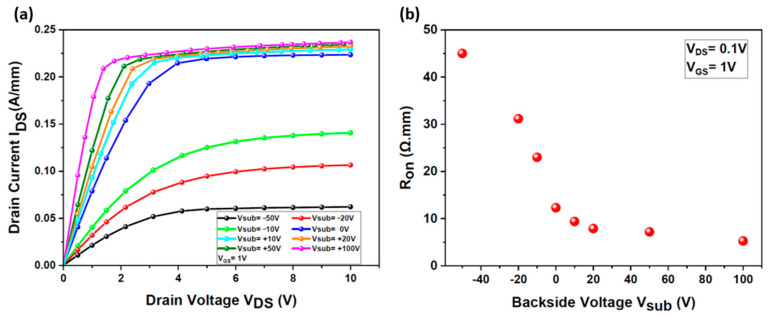
Impact of the applied backside voltage (V_sub_) on the ON-state resistance (R_ON_) of the simulated HEMT: (**a**) I_DS_ (V_DS_) output characteristics of the HEMT for different applied backside voltages (V_sub_) from −50 V to +100 V; (**b**) ON-state resistance variation as a function of V_sub_.

Upon completing the calibration, the epi-structure shown in Figure 2 was employed to analyze the effects of applying high voltage to the backside contact (V_sub_) of the HEMT. The voltage was varied from V_sub_ = −50 V to + 100 V. Figure 4a shows the output characteristics I_DS_(V_DS_) obtained at V_GS_ = 1 V for different applied backside voltage values.

Applying a positive voltage to the backside contact of the HEMT increases the electron sheet density in both the 2DEG channels beneath the gate and the access region. The ON-state resistance (R_ON_), derived from the output characteristics under various backside voltages at V_DS_ = 0.1 V (Figure 4a), is shown in Figure 4b. Notably, R_ON_ decreases from 45 Ω.mm to 5.3 Ω.mm as the applied voltage varies from −50 V to +100 V. This reduction in R_ON_ is attributed to the higher free carrier concentration within the 2DEG, as previously discussed. The backside contact functions as an additional gate, directly influencing the drain current of the device [19].

**Figure 5:**
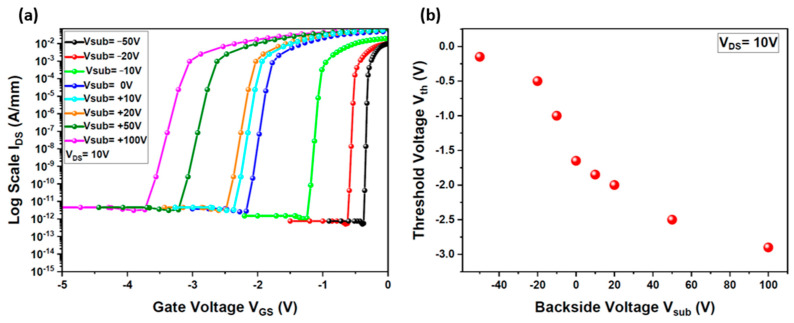
Impact of backside voltage (V_sub_) on the threshold voltage (Vth) of the simulated HEMT: (**a**) I_DS_ (V_GS_) transfer characteristics (log scale) of the HEMT under varying backside voltages (V_sub_) ranging from −50 V to +100 V; (**b**) variation in the threshold voltage as a function of V_sub_.

Another critical parameter to assess is the threshold voltage (Vth) response to the applied backside voltage. Threshold voltage instability is a significant challenge that must be addressed to advance the use of GaN HEMTs in integrated circuits [20]. Figure 5a illustrates the I_DS_(V_GS_) transfer characteristics (log scale) of the HEMT with different applied backside voltages ranging from −50 V to +100 V, while Figure 5b shows the threshold voltage variation as a function of V_sub_. Notably, the current study, conducted under conditions of low trap densities, did not reveal significant hysteresis effects. The threshold voltage (Vth) is determined at I_DS_ = 1 mA/mm. With V_DS_ set to 10 V and V_sub_ at 0 V, the device exhibits a threshold voltage of −1.7 V. However, when the backside voltage is increased to +100 V, Vth shifts negatively to −2.95 V. Conversely, a decrease in V_sub_ to −50 V results in a positive shift in Vth to −0.2 V. This significant threshold voltage instability, induced by variations in the backside voltage, can severely impact the output signal of the gate driver circuit and, consequently, the performance of the high-power vertical device. Clearly, this presents a substantial issue that must be resolved to prevent the lateral device from being affected by the polarization conditions of the vertical one.

### 3.2. Effective Isolation to Mitigate Backside Voltage Influence on the HEMT Device

To counteract the impact of the backside voltage originating from the monolithically integrated high-power vertical transistor on the HEMT device, efficient electrical isolation between the two devices is essential. To achieve this, a highly n-doped GaN layer (n^+^ GaN) is strategically inserted between the epitaxial layers of the two devices. This n^+^ GaN layer, connected to the source contact, is designed to enhance the stability of the HEMT device characteristics. By grounding the n^+^ GaN layer, it acts as a field-blocking barrier, effectively isolating the HEMT transistor from the voltage applied to the backside of the device.

**Figure 6:**
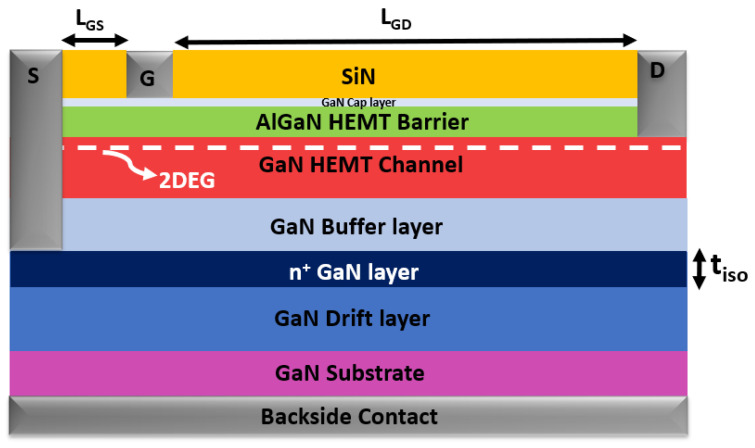
Schematic cross-section of the AlGaN/GaN HEMT featuring backside contact and a n^+^ GaN insulation layer. The n^+^ GaN layer is strategically connected to the source contact, as implemented in the TCAD-Sentaurus simulations.

Figure 6 illustrates the schematic cross-section of the simulated AlGaN/GaN HEMT structure with the added highly doped n^+^ GaN layer. To validate this approach, TCAD simulations were performed with the inclusion of the additional layer, featuring a thickness (t_iso_) of 10 nm and an n-doping concentration of 1 × 10^19^ cm^−3^.

**Figure 7:**
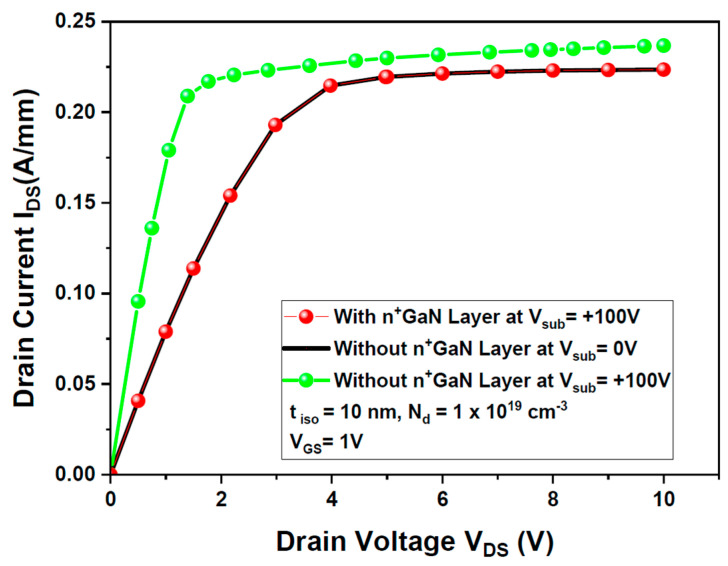
I_DS_ (V_DS_) output characteristics of the simulated HEMT with and without a n^+^ GaN layer at V_sub_ = +100 V applied on the backside contact.

The output characteristics presented in Figure 7 demonstrate that the inclusion of the n^+^ GaN layer effectively shields the device from the influence of the backside voltage. As the backside voltage is increased from 0 V to +100 V, there is no observable change in the output characteristics, indicating successful isolation. The sub-threshold slope (SS) values for the simulated GaN HEMT with and without the isolation layer are 61.5 mV/decade and 72 mV/decade, respectively. The simulated GaN HEMT demonstrates an improved sub-threshold slope (SS) with the isolation layer, achieving 61.5 mV/decade compared to 72 mV/decade without it. This reduction in the SS indicates enhanced gate control and switching efficiency. The contrast between the output characteristics of the HEMT with and without the n^+^ GaN layer at +100 V backside voltage clearly confirms the effectiveness of this isolation technique, ensuring that the HEMT remains unaffected by the high-power device integrated alongside it.

**Figure 8:**
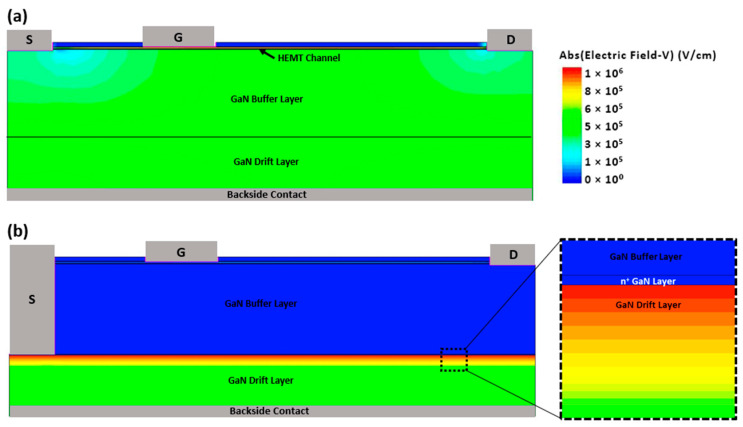
Two-dimensional TCAD-Sentaurus simulation of the HEMT showing the electric field distribution at +100 V (**a**) without the n^+^ GaN layer, and (**b**) with n^+^ GaN layer, highlighting the differences in electric field distribution across the device layers (zoomed view).

Figure 8 illustrates the electric field distribution within the HEMT device both with and without the n^+^ GaN layer. The results demonstrate that the n^+^ GaN effectively blocks the high electric field generated by the backside voltage from reaching the HEMT. Consequently, the 2DEG in the lateral GaN HEMT remains unaffected by the applied backside voltage, ensuring that the device’s performance remains stable and free from degradation.

**Figure 9:**
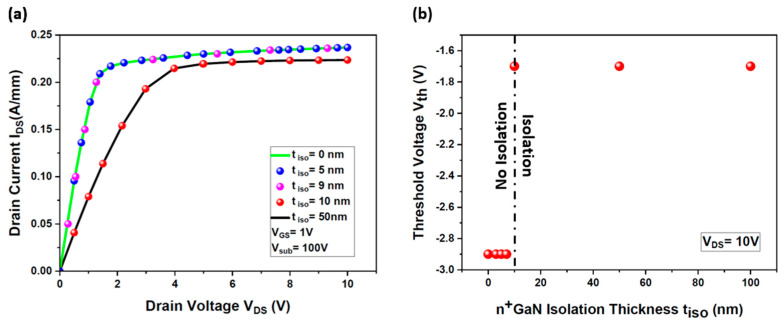
Impact of the thickness of the n^+^ GaN isolation layer (t _iso_) on the threshold voltage (Vth) of the simulated HEMT: (**a**) I_DS_ (V_DS_) output characteristics of the HEMT for different thickness values of the n^+^ GaN layer (t_iso_) at an applied backside voltage V_sub_ = +100 V; (**b**) threshold voltage variation as a function of t_iso_. In these simulations, the doping concentration of the n^+^ GaN isolation layer is N_d_ = 1 × 10^19^ cm^−3^.

Figure 9a unveils the output characteristics of the HEMT device across a spectrum of n^+^ GaN isolation layer thicknesses (t_iso_) doped at 1 × 10^19^ cm^−3^ and ranging from 0 nm to 50 nm. The results irrefutably confirm that robust electrical isolation is attained only when the n^+^ GaN layer achieves a pivotal thickness of 10 nm. Figure 9b further reinforces that the Vth of the HEMT device remains unwaveringly stable at −1.7 V, even under the duress of a formidable backside voltage of up to +100 V, provided that the n^+^ GaN isolation layer upholds this crucial 10 nm minimum thickness. Accordingly, this 10 nm thickness will be rigorously employed in forthcoming simulations to delve into the profound influence of n^+^ GaN layer-doping on the isolation capabilities of the device.

**Figure 10:**
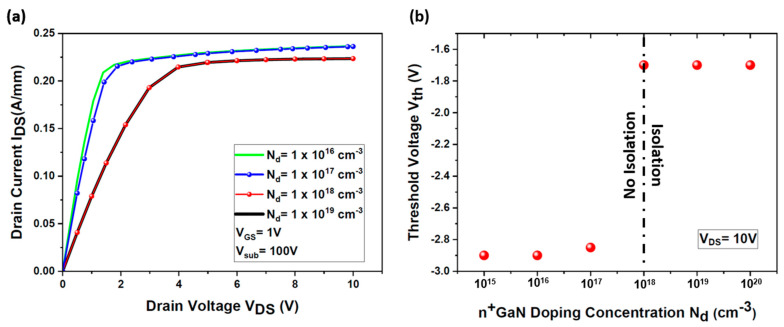
Impact of the doping concentrations of the n^+^ GaN layer (N_d_) on the threshold voltage (Vth) of the simulated HEMT: (**a**) I_DS_ (V_DS_) output characteristics of the HEMT for different doping concentration values of the n^+^ GaN layer (N_d_) at an applied backside voltage V_sub_ = +100 V; (**b**) threshold voltage variation as a function of N_d._ In these simulations, the n^+^ GaN isolation layer thickness is equal to 10 nm.

We have rigorously examined the isolation efficacy of the n^+^ GaN layer as a function of its doping concentration, spanning from N_d_ = 1 × 10^16^ cm^−3^ to N_d_ = 1 × 10^19^ cm^−3^. As depicted in Figure 10a, it becomes evident that doping concentrations below 1 × 10^18^ cm^−3^ render the layer inadequate in mitigating the high electric field emanating from the backside voltage, thereby failing to secure the necessary isolation for the HEMT device. Remarkably, the threshold voltage (Vth) exhibits unwavering stability at Vth = −1.7 V, even under the imposition of a substantial backside voltage of +100 V, provided the doping concentration (N_d_) of the n^+^ GaN isolation layer meets or exceeds the critical threshold of 1 × 10^18^ cm^−3^, as illustrated in Figure 10b. This outcome underscores the quintessential role of the n^+^ GaN layer’s precise doping specifications in fortifying the HEMT device against the deleterious effects of backside voltage. The strategic selection of doping levels and layer thicknesses emerges as paramount in engineering the requisite electrical isolation between the vertically and laterally integrated devices.

The isolation capability of the n^+^ layer depends on the quantity of charges present in this layer. These charges will screen the electric field induced by the voltage applied to the device’s backside electrode. Thus, for the same V_sub_, different thicknesses and doping levels will be effective in stabilizing the device’s Vth. For a thin n^+^ layer, the doping must be high enough to achieve our objective. On the other hand, for a thick n^+^ layer, the doping can be reduced while maintaining the good isolation behavior of this layer.

## 4. Conclusions

The monolithic integration of vertical and lateral devices on a single chip is contingent upon achieving robust electrical isolation between these components. To address this, we employed a highly doped n-type GaN layer (n^+^ GaN) with a donor concentration of 1 × 10^19^ cm^−3^ and a thickness of 10 nm, simulated using TCAD-Sentaurus. The electrical output characteristics, I_DS_ (V_DS_), reveal that without this critical isolation layer, the backside contact effectively acts as a back-gate, destabilizing the device’s threshold voltage. By incorporating the n^+^ GaN layer within the HEMT epi-structure, we successfully neutralize this destabilization, ensuring that the output characteristics remain stable across varying backside voltages. This innovative solution not only resolves a significant obstacle but also paves the way for the reliable monolithic integration of vertical and lateral devices on a unified chip architecture.

## Figures and Tables

**Table 1:** Thicknesses and donor doping concentrations of the different layers for the simulated HEMT device by TCAD-Sentaurus.

Layer	Thickness (µm)	Doping Concentration (cm^−3^)
GaN Cap	0.002	1 × 10^16^
AlGaN	0.02	1 × 10^16^
n-GaN HEMT Channel	0.05	1 × 10^16^
n-GaN Buffer	0.5	1 × 10^16^
n-GaN Drift	0.3	1 × 10^16^
n^+^ GaN Isolation	0.01	1 × 10^19^

## Data Availability

The data presented in this study are new and have not been previously published.
